# Multicomponent interventions designed to support adherence to guideline-recommended therapy in patients with peripheral artery disease: A scoping review

**DOI:** 10.1177/1358863X251315071

**Published:** 2025-03-13

**Authors:** Smaragda Lampridou, Tania Domun, Javiera Rosenberg, Rachael Lear, Alun Huw Davies, Mary Wells, Gaby Judah

**Affiliations:** 1 Vascular Surgery Department, Imperial College Healthcare NHS Trust, London, UK; 2 Department of Surgery & Cancer, Faculty of Medicine, Imperial College London, London, UK; 3 Nursing Directorate, Imperial College Healthcare NHS Trust, London, UK

**Keywords:** peripheral artery disease (PAD), guideline-directed medical therapy, medication adherence, behavior change techniques

## Abstract

Adherence to guideline-recommended therapies for peripheral artery disease (PAD), including pharmacotherapy (antiplatelet, lipid-lowering, and antihypertensive agents) and lifestyle modifications (smoking cessation, diet, weight management, and physical activity) remains low. Though single-component interventions targeting smoking cessation, exercise, or medication adherence show some efficacy, comprehensive multicomponent interventions are vital for addressing the complexity of PAD management. This review systematically synthesized multicomponent interventions for patients with PAD. A systematic search was conducted in Embase, MEDLINE, Cochrane Library, APA PsycINFO, CINAHL, Web of Science Core Collection, ProQuest, and Google Scholar to identify primary research describing multicomponent interventions supporting PAD treatment adherence, published between 2007 and 2024. A narrative synthesis was reported using the Template for Intervention Description and Replication (TIDieR) checklist and the behavioral change techniques (BCT) taxonomy. Out of 15 studies (2462 patients, 60.4% men) included in this review, only two addressed all guideline-recommended treatment aspects. Key intervention components included structured exercise (12/15) and education programs (10/15). Most interventions were delivered by multidisciplinary teams in hospital settings over 3 months. Only one study employed behavioral theory in its development, and most interventions (13/15) focused on the BCT ‘instructions on how to perform a behavior’ rather than diverse BCTs. No interventions significantly increased adherence to all PAD therapies. Few studies measured the intervention’s impact on adherence, making it difficult to determine effective intervention characteristics. Most interventions lacked behavioral science approaches and were not designed to address specific adherence determinants. Future interventions should incorporate these elements to effectively address patients’ needs. **Open Science Framework Registry ID: osf.io/7xqzj**

## Background

Lower-extremity peripheral artery disease (PAD) is a chronic condition caused by peripheral artery atherosclerosis, reducing blood flow to the legs.^1^ Global prevalence has increased, with 113 million individuals aged 40 years and above affected in 2019.^2^ Patients with PAD, compared to healthy individuals, face up to a 15-fold increase in mortality risk, predominantly attributable to cardiovascular events, highlighting the need for effective management.^3–5^

Adherence to guideline-recommended therapy for lower-extremity PAD, including antiplatelet or antithrombotic medications, lipid-lowering agents, antihypertensives (in the case of hypertension), smoking cessation, and lifestyle modifications (healthy diet, weight management, and exercise),^5–7^ can reduce major cardiovascular and limb events by 40% and extend life expectancy by 6 years.^8,9^ Despite these benefits, adherence to PAD treatment remains poor,^10–14^ posing significant risks for patients.^9,15^ A recent review, mainly using prescription records, reported antiplatelet adherence between 27.5% and 96.3% and lipid-lowering medication adherence from 23.5% to 92.0%.^14^ This variability may be due to prescription records indicating prescription collection or receipt but not actual medication intake.^14,16,17^ Notably, over half of patients with PAD discontinue statins within the first year.^10–12^ International registry data indicate over 80% of patients with PAD are current or ex-smokers, and only 10% participate in supervised exercise therapy (SET).^10,18^ Our previous study, using validated self-reported adherence methods, revealed only a third of patients had high adherence to antiplatelets and statins, 25% still smoked, and only 17.1% completed SET.^13^ Although validated self-reported measures are considered more reliable for assessing adherence, they have limitations, including potential overestimating of adherence by patients and recall bias.^19,20^

Improving adherence is crucial to improving clinical and patient outcomes.^15^

Research on interventions supporting patients with PAD with their care is growing, but most target a single behavior like exercise or smoking cessation.^21,22^ As health behaviors often co-occur, focusing on multiple behaviors may better support the overall management of patients living with chronic conditions^23,24^ like PAD. However, addressing multiple versus a single behavior in one intervention poses distinct challenges compared to addressing a single behavior. A few interventions have incorporated a holistic approach addressing all guideline-recommended therapies for PAD, but the optimal content and delivery of effective multicomponent interventions is unclear.

A successful single-component study to improve walking capacity in patients with PAD used behavior change theories and behavioral change techniques (BCTs) to design and describe the intervention.^25^ The BCT Taxonomy v1^26^ is a validated classification system categorizing 93 behavior change techniques into 16 overarching categories, offering clear definitions and examples of behavioral change ‘active ingredients’. This standardized approach aids the classification of interventions and enables the replication of techniques in research and practice, regardless of frameworks or theories employed to develop the interventions, or different ways of delivering the interventions.^26^

This scoping review aimed to systematically synthesize evidence on multicomponent adherence interventions for patients with PAD, focusing on intervention components, theoretical frameworks, and implementation barriers.

### Review questions

1. What multicomponent interventions exist to support adherence to medications (antiplatelet, lipid-lowering, antihypertensive), smoking cessation, diet, and/or physical activity in patients with PAD?
2. What are the key characteristics and BCTs of these interventions? Are interventions grounded in theoretical frameworks or designed to address specific nonadherence determinants?
3. Which interventions are effective in promoting adherence?
4. How is adherence defined and measured in these interventions?
5. What facilitates or impedes the implementation of these interventions in clinical practice?

## Methods

This review was conducted following the Joanne Briggs Institute (JBI) methodology for scoping reviews^27^ and in line with the Preferred Reporting Items for Systematic reviews and Meta-Analyses extension for Scoping Reviews (PRISMA-ScR).^28^ The protocol was registered with Open Science Framework (registry ID: osf.io/7xqzj).

### Search strategy

The JBI three-phase search approach was used. With the assistance of a senior healthcare librarian, key terms from relevant article titles, abstracts, and indexes informed the search strategy (Supplementary Table 1). This approach was adapted for Embase (Ovid), PsycINFO (Ovid), Cochrane Library, CINAHL (EBSCOhost), and Web of Science Core Collection. To reduce publication bias, clinical trials registries (clinicaltrials.gov) and grey literature sources (ProQuest Dissertations and Theses Global and Google Scholar) were searched using the keywords “peripheral artery disease” and “patient compliance” or “treatment adherence.” References from included studies were also screened for additional papers.

### Eligibility criteria

We included studies of adult participants (⩾ 18 years old), regardless of sex, ethnicity, comorbidity, or form of PAD (intermittent claudication, asymptomatic PAD, or chronic limb-threatening ischemia [CLTI]). Studies of patients with neurogenic claudication or in which PAD data could not be isolated were excluded. Studies were included if the intervention targeted two or more health behaviors; for example, medication adherence and physical activity. Single-component interventions, or those that targeted multiple aspects of one behavior (i.e., multiple forms of physical activity), were excluded. Interventions could be psychological, behavioral, educational, pharmacological, or involve self-management support, delivered digitally or in-person by any personnel. Studies from any location or healthcare setting were included, with no sample size or language limits. As the first PAD guidelines were published in 2007,^29^ we included papers published between 2007 and the present. The initial search was on January 14, 2024; a repeat search was undertaken on June 12, 2024. We included peer-reviewed and nonpeer-reviewed primary research (quantitative, qualitative, and mixed methods), intervention protocols, and studies (qualitative or surveys) evaluating the feasibility, fidelity, and acceptability of interventions. Studies that informed the development of interventions (e.g., surveys, qualitative research, or co-production) were also considered if details about the developed interventions could be extracted. Reviews, meta-analyses, opinion articles, and conference abstracts or posters were excluded.

### Evidence selection

All references were imported into the Covidence software (https://app.covidence.org/sign_in). After automatic de-duplication, any remaining duplicates were manually removed. Three reviewers with cardiovascular (SL), public health (TD), and psychology (JR) backgrounds independently screened papers for inclusion in two stages. First, all titles and abstracts were screened by two reviewers (SL and TD). Second, the lead reviewer (SL) screened 100% of the papers at the full-text stage, and each of the other two reviewers (JR and TD) screened 50% of the papers. The reviewers were blinded to each other’s choices. Conflicts were resolved through a discussion, and an additional independent reviewer was consulted when required (GJ). When necessary, authors were contacted via email to retrieve full text, missing, or additional data. Reasons for exclusion were recorded.

### Data extraction and synthesis

Data were extracted from included papers by two independent reviewers using a template designed for this study within the Covidence software’s extraction tool. Two reviewers (SL and TD) pilot-tested the extraction tool with three studies. The tool was used to extract: study characteristics (title, authors, year of publication, aim, study design, country of origin, and setting), participant characteristics (age, sex, ethnicity, comorbidities), and key results. Additionally, the Template for Intervention Description and Replication (TIDieR) framework was adapted to describe the characteristics and details of the interventions.^30^ Finally, the content of each intervention was mapped onto the BCT Taxonomy v1^26^ to detail and categorize the BCTs used. For this review, every BCT reported in an intervention was coded (interventions can include multiple BCTs) by two independent researchers (SL and TD) and cross-checked by a third reviewer (JR). All reviewers were trained in the taxonomy. To increase validity, reviewers were blinded to each other’s extracted data until the consensus stage, with conflicts resolved by discussion or a third reviewer (JR/GJ).

Assessing the quality of the papers was not within the scope of this review.^31^

A narrative synthesis was conducted, following JBI guidance, and the characteristics of included studies were presented in tabular and narrative formats. The TIDieR framework guided the intervention synthesis.^30^

## Results

**Figure 1.**
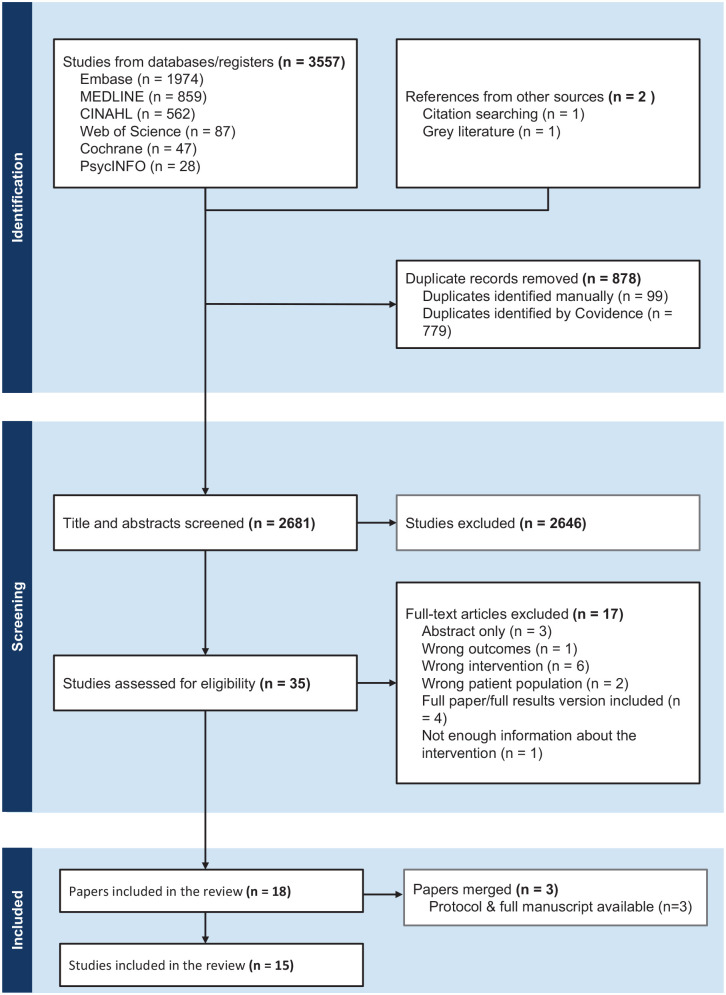
PRISMA flow chart.

The initial search identified 3557 studies, of which 878 were removed as duplicates (Figure 1). At the full-text stage, three relevant studies were identified but only had abstracts available.^32–34^ These studies were excluded after unsuccessful efforts to contact authors for full texts. After screening, 18 papers were identified for data extraction and synthesis. Of these, three studies had multiple relevant publications.^35–37^ Separately published protocols and results were merged during data extraction. Hence, 15 studies were included in the review.^35–49^ Around half (7/15) were undertaken in Europe,^36,37,40,43,45,47,48^ three in the UK,^39,42,46^ two in the USA,^44,49^ two in Canada,^35,41^ and one in Latin America.^38^ A summary of included studies is presented in Supplementary Table 2.

### Participant characteristics

Included studies encompass a total of 2462 patients with PAD (60.4% men). Most studies included patients with stable claudication, both before^35,37,46^ or after vascular surgery,^41,43,48^ though some did not report the patients’ previous vascular surgery history.^38,39,45,47,49^ Patients with severe claudication and CLTI were excluded from five studies.^35,39,40,42,48^ The selection criteria for each study are presented in Supplementary Table 3.

### Adherence definition and assessment

All included studies described activities related to adherence; however, most studies did not define the term. Adherence was often described as ‘compliance with the medical plan,’ ‘risk factor management,’ ‘commencing medications,’ and ‘exercise class attendance’. Only Haile et al.’s study provided a clear definition of medication adherence as ‘the proportion of days covered calculated by dividing the number of available dispensed doses (registry data) by the number of days the patient was prescribed the medication (data from medical records)’.^36^

Assessment of adherence was also underreported. Most studies (9/15) used self-reported methods to assess adherence.^35,36,38,39,41–43,45,47^ For medication adherence, Davins Riu et al.’s study used a validated questionnaire (Morisky–Green test),^45^ whereas Rosenberg and Behrendt used prescription records.^43^ Two studies employed carbon monoxide assessment to evaluate smoking cessation,^39,46^ and only Cucato et al.’s study used a pedometer to monitor exercise.^39^ Fallon et al.’s study noted standardized measures to track risk factor management, including smoking cessation; however, these measures were not described.^48^

### Rationale and theory for intervention

Most studies (10/15) did not use a theory or framework for developing their interventions.^35,40–48^ Only two studies used validated frameworks: the FASTIC study employed the Medical Research Council (MRC) complex intervention framework^36^ and Mendez et al.^38^ used the Contextualized Instructional Design model. The CIPIC Rehab intervention was the sole study to incorporate a behavioral theory (Social Cognitive-Behavioral Theory^37^) described only in the study protocol.^50^ Two studies utilized care pathways: Person-Centered Care in the FASTIC study^36^ and the Chronic Care Model in Lovell et al.’s study.^49^ Cucato et al.’s study uniquely incorporated co-production elements, specific BCTs, and targeted adherence barriers, such as financial limitations and travel distances.^39^

### Intervention characteristics

All interventions focused on patients with PAD, rather than family, caregivers, or healthcare providers. Study designs included two protocols for randomized controlled trials (RCTs),^39,47^ one intervention development,^38^ one quasi-experimental study,^49^ five RCTs,^35–37,43,45^ and six observational studies.^40–42,44,46,48^

#### Targeted behaviors

Interventions focused on different targeted behaviors required for guideline-recommended therapy, including exercise/exercise class attendance, adoption of optimal nutrition habits, medication adherence, smoking cessation, alcohol reduction, and weight management (Supplementary Tables 4 and 5). We acknowledge that weight reduction is an outcome of behavior, rather than behavior itself, and can result from diet and/or exercise. However, in the included studies, weight management was presented as a behavior. For this review, diet and exercise will be examined as behaviors, and weight management as an outcome of the behavior.

Only two interventions covered all recommended treatment aspects^47,48^ (Supplementary Tables 4 and 5). Two interventions targeted two different aspects of the treatment plan: Hussain et al.’s study addressed medication adherence and smoking cessation^41^; Mendez et al. focused on medication adherence and exercise.^38^ Physical activity was the most prevalent target behavior, reported across 12 interventions.^35–40,42–45,47,48^

#### Behavioral change techniques

The application of BCT Taxonomy v1 identified 21 different BCTs used in the interventions (Supplementary Table 6). The number of BCTs coded in each study ranged from three^41,42,46,48^ to 12 BCTs,^49^ with a mean of six. The most used BCT was ‘instructions on how to perform a behavior’ (13 studies); followed by ‘self-monitoring of behavior’ (seven studies), and ‘goal setting (behavior)’ (five studies). The least frequently used were ‘conserving mental resources,’ ‘monitoring of behavior by others without feedback,’ ‘review of outcome goals,’ and ‘feedback on outcomes of behavior’ (each used in one study). We cannot report the proportion of effective BCTs due to the limited statistical power in the included studies.

Considering the total BCTs used for each target behavior, most were used to support physical activity/exercise uptake (18 BCTs) (Supplementary Table 6). The lack of information provided in one protocol study^42^ meant it was particularly challenging to map some BCTs for each target behavior.

#### Provider of the intervention

Interventions were delivered by various healthcare professionals and often adopted a multidisciplinary approach (9/15 studies). Nurses and physiotherapists were most frequently involved. Four studies reported referral to specialist professionals for specific needs, such as diabetes specialist nurses or smoking cessation specialists.^36,41,46,48^ Specialized training for providers was reported in four studies, covering motivational interviewing,^35,36,39^ person-centered care,^36^ and best medical therapy.^43^ Besides healthcare professional input to support adherence, four studies also incorporated elements of patient self-management for exercise and risk factor modification.^35,36,39,46^

#### Mode of delivery and location

Seven interventions were exclusively face-to-face (F2F), primarily in hospitals^40,41,46,48,49^ or community settings.^42,44^ Two studies were delivered in both primary and secondary care settings.^37,47^ Four studies^35,36,39,43^ incorporated both hospital F2F elements and home-based, remote monitoring, including remote exercise classes^35,39,43^ or remote follow-up calls.^36^ Two interventions were app-based,^38,45^ with one delivered entirely virtually,^38^ and the other including both virtual and clinical F2F appointments.^45^

#### Duration and time-points

The duration, intensity, and dose of the interventions varied. Intensive programs included 12-week exercise and education sessions held multiple times weekly.^35,37,39,42,46,47^ Less intensive approaches featured nurse-led cardiovascular risk management with follow ups and telephone support ranging from weekly^43^ to biannual contacts.^36^ Höbaus et al.’s study featured the longest follow-up period, with clinical appointments every 6 or 12 months for up to 5 years.^40^

Interventions were delivered at different times in the patient’s disease journey, with most targeting postsurgery patients. Only Hatfield et al.’s study was specifically targeted to newly diagnosed patients.^46^ In two studies, patients were recruited before revascularization, but the intervention was delivered postrevascularization.^36,44^

### Tailoring of the interventions

Around half of the interventions (8/15) included elements of personalized care; however, the level of tailoring was underreported and varied. Studies typically reported offering a personalized assessment and risk factor management plan, dependent on patient interactions with the intervention^37,45^ or risk stratification.^36,44,48,49^ Additionally, three studies offered a personalized exercise plan based on the patient’s exercise performance.^35,42,48^

### Evaluation of interventions

All 12 primary studies evaluated the impact of the interventions, but none effectively increased adherence to all targeted behaviors (Supplemental Table 3).

Seven studies examined the effect of interventions on medication adherence.^36,40–43,45,46^ Six studies showed a positive trend,^40,41,43,45,46^ with two reporting 100% adherence at the end of follow up;^43,46^ however, the statistical significance of these results was not provided. Davins Riu et al.’s study noted short-term improvement at 6 months (83.8% vs 69.0%; *p* = 0.031), but this was not maintained at 12 months (*p* = 0.317).^45^ Haile et al.’s study was unsuccessful in improving adherence to secondary preventive medication (79% vs 82% adherence to lipid-modifying agents; *p* = 0.464).^36^ Sutton et al.’s study reported all patients were prescribed statins by the end of the program, but adherence rates were not provided.^44^ Although a further three studies incorporated elements to support medication adherence, they did not report outcomes.^37,48,49^

Seven reported smoking cessation,^35,41–43,46,48,49^ with quit rates ranging from 6%^48^ to 40%.^35^ Only Hussain et al.’s observational study noted a statistically significant effect of the intervention on smoking cessation.^41^ Four studies noted a slight reduction in cigarettes smoked.^40,42–44^ Additionally, three studies^36,37,45^ found more patients quit in the intervention arm compared to control, but the results were not statistically significant, indicating no effect of the intervention on smoking cessation.

Six studies examined the impact of the interventions on exercise/physical activity.^36,37,43–45,48^ Only two interventions were found to be effective in increasing walking capacity and exercise uptake.^37,48^ In the CONTECI study, although more patients exercised in the intervention group (21.4%) than those in the control group (14.7%), the results were not statistically significant (*p* = 0.125).^45^

Moreover, two studies found that fewer than 25% of patients met the exercise guideline-recommended goal.^43,44^ In Haile et al.’s study, initially, significantly more control group patients had unlimited walking distance (75 of 88 vs 58 of 97; *p* = 0.001), but this difference disappeared after 1 year (56 of 95 vs 61 of 93; *p* = 0.370).^36^

Of eight studies incorporating both an exercise and a healthy eating component, seven examined the intervention’s impact on weight reduction. Of those, only Siercke et al.’s study was effective in supporting patients to achieve a healthy diet at 6 months’ follow up^37^; and Hussain et al.’s in achieving optimal BMI.^41^ Three other interventions positively impacted weight reduction,^35,42,43^ yet the results were not statistically significant. Lastly, two studies indicated no significant change in weight management.^48,49^

### Acceptability of interventions

Only three studies^36,37,39^ reported assessing acceptability from either patients’ or staff’s perspectives. Cucato et al.’s study protocol outlined intentions to assess intervention acceptability through semistructured patient interviews.^39^ In the FASTIC study,^36^ 17 patients who completed the intervention participated in qualitative interviews, highlighting the intervention’s perceived value.^51^ The CIPIC Rehab study also reported conducting a qualitative evaluation of interventions, but the publication detailing these findings is pending.^50^ Only one study (CONTECI telehealth intervention) included an economic evaluation, showing a reduction in healthcare costs, mainly due to less frequent outpatient (95.95% reduction; *p* < 0.000) and emergency department patient visits (intervention 0.19 vs control 0.017; *p* = 0.017).^45^

## Discussion

This scoping review explored multicomponent interventions to support treatment adherence in patients with PAD. Developing comprehensive, personalized interventions is a challenge. Behavioral science approaches have, so far, been underused. As there is limited consensus on defining, measuring, and addressing adherence, and the components that adherence interventions should incorporate, it is difficult to compare studies. None of the included studies effectively improved adherence to all targeted behaviors and only one reported patient intervention acceptability.

The review concurred with a previous review on PAD exercise therapy,^18^ noting that most studies lacked consensus on adherence definition, often describing adherence as risk factor management or exercise class attendance. Most studies relied on self-reported adherence methods (e.g., exercise diaries), which can overestimate adherence due to recall bias and selective reporting.^16,17,52^ Consequently, self-reporting may not reliably detect poor adherence.^52^

Given that all included interventions have multiple components, they meet the MRC definition of a complex healthcare intervention,^53^ yet only one study incorporated the MRC framework in its development.^36^ The MRC framework and current literature highlight the importance of theory-driven behavioral change interventions,^54,55^ yet only one study^37^ employed such theory; and another employed specific BCTs to address adherence barriers.^39^ The most common BCTs used were ‘instructions on how to perform a behavior’ followed by ‘self-monitoring of behavior’ and ‘goal setting (behavior)’; however, conclusions cannot be drawn on which BCTs were associated with intervention effectiveness. This, alongside the lack of behavioral theory in intervention design, highlights a gap in developing interventions using behavioral science to address identified determinants of behavior and change behavior using effective BCTs.

Simply providing information is insufficient to change behavior.^56^ Self-regulatory techniques, like self-monitoring and goal setting, have proven effective in enhancing physical activity in chronic diseases, like diabetes.^57,58^ Nevertheless, patients’ beliefs about their condition and treatment influence their management and adherence.^59^ Therefore, treatment information should be tailored to address patients’ beliefs, while aligning with evidence-based recommendations.^60^ In our review, many interventions (8/15) included personalized care elements; however, the level of tailoring was underreported and varied, and it is hard to assess whether the intervention’s success was linked to the personalized components. Personalized interventions are more effective in changing behavior,^61^ empowering patients and enhancing satisfaction and self-management.^62^ Therefore, examining the effect of personalized interventions on adherence in patients with PAD is essential.

The reviewed studies incorporate lifestyle and risk factor management strategies. A recent meta-analysis identified that focusing on multiple behaviors simultaneously is effective in chronic disease management.^63^ Such interventions can increase physical activity, improve dietary habits (increased fruit and vegetable intake, decreased fat intake), reduce alcohol consumption, and enhance medication adherence.^63^ Positive effects have been observed immediately postintervention and during follow ups.^63^

This review found variability in who delivered the interventions, with limited details on training required for delivering. Most studies involved multidisciplinary teams, with nurse specialists and physiotherapists more frequently mentioned. Similar findings were identified in reviews of interventions for patients with multimorbidity.^64^ In common with other reviews, examining interventions in patients with chronic diseases, such as stroke and venous ulceration,^65–67^ it was unclear who was responsible for the delivery of each component. Several studies incorporated self-management elements, vital in chronic illness care, to improve clinical outcomes, quality of life, and reduce hospitalizations.^68,69^ A recent randomized trial indicated that a nurse-led medication self-management intervention increased short-term medication adherence and health outcomes in older multimorbid patients.^70^

Lastly, interventions were often delivered in a hospital setting to stable patients with PAD. Only one intervention in this review was delivered to newly diagnosed patients with PAD,^46^ despite evidence from our previous study highlighting that newly diagnosed patients perceive their disease as less chronic and threatening, leading to lower adherence.^13^ This suggests a need for further evidence and debate between clinicians, patients, and researchers regarding the optimal time on the patients’ journey to deliver such interventions.

### Strengths and limitations

This review is the first to synthesize available evidence on multicomponent interventions to support treatment adherence in patients with PAD. It followed the JBI scoping review and PRISMA-ScR guidelines, using a checklist to report the appropriate information. Database selection and search terms were meticulously planned and guided by consultations with an independent senior healthcare librarian, identifying papers from both journals and grey literature to ensure comprehensive coverage.

Although this review was conducted systematically, several limitations exist. Despite efforts to ensure a robust search strategy, some relevant studies may have been missed. A component network meta-analysis, which estimates the effects of individual components within multicomponent interventions, was not feasible given the scoping nature of this review.^71^ Hence, we could not assess individual intervention components’ effectiveness or their synergistic or antagonistic interactions.^72^ Similarly, a detailed analysis of study outcomes and risk of bias could not be conducted.

Additionally, bias may have been introduced due to the lack of standardized adherence terminology during the search and screening processes. The main limitation of this review is the heterogeneity of included studies and intervention components, making it challenging to fully answer the research questions. Moreover, insufficient information in the included studies hindered detailed BCT analysis, which can affect the validity of results. In some studies, assessing which BCTs specifically targeted each behavior was difficult. Although reviewers undertook BCT training and ‘matched’ their coding, BCTs may have been underreported. Lastly, most of the included studies were not statistically powered to improve adherence. This limitation makes it challenging to draw definitive conclusions on the effectiveness and implementation of these interventions in clinical practice.

### Future research implications

This review highlights the need to develop more structured, theory-driven, large-scale multicomponent studies targeting vascular patients. Future studies could benefit from utilizing intervention development frameworks, such as the MRC Framework for developing and evaluating complex interventions to address the multicomponent nature of the interventions, and consider target-user satisfaction and acceptability. Future interventions should combine disease education, exercise sessions, and self-management support, while integrating multiple BCTs known for their effectiveness, to give the best chances for success. Studies should report on the BCTs used, intervention feasibility, and fidelity. This approach will allow focused evaluation of what works best in particular adherence areas and inform future, more comprehensive trials later.

The introduction of complex, multicomponent interventions for vascular surgery patients should be sequential, starting with pilot trials in single centers to fine-tune interventions and assess feasibility. These can inform larger multicenter trials, which provide generalizability and diverse patient representation. The intervention should always be tested against a control group, in which standard care is provided, to assess the added value of multicomponent approaches from usual care practices. Specific trial design will depend on the primary outcomes. Based on the results of this review, we suggest adherence rates, measured using validated methods, to all guideline-recommended treatments are considered as primary outcomes. Secondary outcomes should also include quality of life and cardiovascular risk reduction. Most RCTs in this review were pilot studies and were underpowered. Hence, it is important that future studies are appropriately powered, and the sample size calculated based on the relevant primary outcomes. Future studies should also target patients with PAD at various stages, ensuring diversity in age, sex, ethnicity, and comorbidity profiles. A minimum intervention duration of 12 weeks is recommended, followed by an extended follow-up period to monitor the sustainability of behaviors. A multidisciplinary team, including professionals from different specialties involved in managing patients with PAD, should also be utilized, with various professionals offering their expert advice to support better disease management.

## Conclusion

Our review highlights that only a few multicomponent interventions target all behaviors, limiting holistic support for patients with PAD. None of the included studies effectively improved adherence to all aspects of the guideline-recommended plan and not enough studies measured the intervention’s impact on adherence, hindering recommendations on effective intervention characteristics. Hence, further research is needed to accurately assess their impact. Most studies lacked robust theoretical frameworks or behavioral change methods. As education alone is insufficient in changing behavior, future research should focus on other behavioral change techniques, including promoting self-management, goal-setting, and problem-solving. The growing interest in personalized interventions is hindered by insufficient information on their implementation. To effectively support patients with PAD in managing their complex treatment plan, robust, evidence-based, behavioral, and personalized multicomponent intervention strategies to support patients with PAD with treatment adherence are needed.

## Supplemental Material

sj-pdf-1-vmj-10.1177_1358863X251315071 – Supplemental material for Multicomponent interventions designed to support adherence to guideline-recommended therapy in patients with peripheral artery disease: A scoping review Supplemental material, sj-pdf-1-vmj-10.1177_1358863X251315071 for Multicomponent interventions designed to support adherence to guideline-recommended therapy in patients with peripheral artery disease: A scoping review by Smaragda Lampridou, Tania Domun, Javiera Rosenberg, Rachael Lear, Alun Huw Davies, Mary Wells and Gaby Judah in Vascular Medicine
