# Supplementary material for: Multicomponent interventions designed to support adherence to guideline-recommended therapy in patients with peripheral artery disease: A scoping review
Source: Vasc Med. 2025 Mar 13;30(3):343–52. doi: 10.1177/1358863X251315071 (PMC12171072; doi:10.1177/1358863X251315071)
Supplement: sj-pdf-1-vmj-10.1177_1358863X251315071 – Supplemental material for Multicomponent interventions designed to support adherence to guideline-recommended therapy in patients with peripheral artery disease: A scoping review [file sj-pdf-1-vmj-10.1177_1358863X251315071.pdf]

Supplementary Material

Table 1. Medline (Ovid) Search strategy

Search	Query	Records retrieved
#1	Peripheral Arterial Disease/ OR (Peripheral Arter* adj2 Disease*).mp. OR Intermittent Claudication/ OR claudication.mp. OR PAD.mp.	60,859
#2	"treatment adherence and compliance"/ OR Patient Compliance/ OR Treatment Refusal/ or Patient Dropouts/ OR exp Attitude to Health/ OR Patient Satisfaction/ OR medication adherence/ OR patient participation/ OR (Dropout or drop-out or drop out).mp. OR Uptake.mp OR Adhere*.mp.	1,162,793
#3	Educat*.mp. OR (patient-education or "patient education").mp. OR ("patient cent*" or patient-cent*).mp. OR (patient-focus* or "patient focus*").mp. OR (“management plan" or management-plan).mp. OR Behavio?r*.mp. OR Cognitive Behavio?ral Therapy/ or Motivational Interviewing/ OR "behav* intervention*".mp. OR Intervention*.mp. OR Intervention* study or Complex intervention*.mp. OR Health program*.mp. OR Health promotion/ OR "follow-up*".mp. OR “follow-up*” adj3 communit* OR “Follow-up” adj3 care OR Exercise Therapy/ OR "exercise program*".mp. OR Support program*.mp. OR behav* change.mp OR Nurs* led.mp. OR nurs* based.mp. OR telephone based.mp. OR (phone adj2 "follow up").mp. OR Telehealth.mp. OR mHealth.mp. OR community based.mp. OR Multidisciplinary.mp. OR multicomponent.mp. OR multifactorial.mp. OR (rehab* adj2 program*).mp.	5,862,966
#4	#1 AND #2 AND #3	1049
Limit added for papers published between 2007- present		848

Table 2. Summary of included study characteristics

Study	Primary outcome	Setting & Study design	Study population			
			**Number & Gender n (%) male**	**Age (mean ± SD)**	**PAD stage**	**Comorbidities n (%)**
**Coca-Martinez M et al., 2023 [35]**	Feasibility and safety of the intervention	Secondary care<br><br>Single-blind, 2-arm, parallel, pilot-RCT	n=24<br><br>Gender<br>Intervention<br>6 (50)<br><br>Control<br>4 (33.3)	Intervention<br>71.7 ± 7.4<br><br>Control<br>69.5 ± 8.1	Pre-vascular intervention /surgery *, stable, moderate to severe claudication.	Intervention<br>IHD 5 (41.7), HTN 10 (83.3), T2DM 7 (58.3), COPD 2 (16.7), CKD 2 (16.7), Obesity 4 (33.3)<br><br>Control<br>IHD 5 (41.7), HTN 11 (91.7), T2DM 4 (33.3), COPD 2 (16.7), CKD 2 (16.7), Obesity 7 (58.3)
**Mendez CB et al, 2019 [38]**	N/A - intervention development	Secondary care<br><br>Intervention development	N/A – intervention development	N/A – intervention development	No mention of disease stage	N/A – intervention development
**Cucato G et al 2022 [39]**	Feasibility and acceptability of the intervention	Secondary care<br><br>Protocol for a pilot RCT	Target recruitment: 60<br><br>Gender<br>N/A - protocol	N/A - protocol	Non-severe PAD or CLTI	N/A - protocol
**Haile ST et al., 2023 [36]**	Medication adherence	Secondary care<br><br>Non-blinded RCT	n=204<br><br>Gender<br>Intervention<br>59 (60)<br><br>Control<br>51 (49)	Intervention<br>age median (IQR) = 71 (66 to 76)<br><br>Control<br>age median (IQR)= 72 (69 to 77)	Patients with IC post vascular intervention /surgery *	Intervention<br>IHD 34(34), HF 4(4),<br>HTN 83 (84), COPD 17 (17), CVA/TIA 15 (15), CKD 1 (1), T2DM 32 (32), Previous vascular intervention 33 (33)<br><br>Control<br>IHD 31 (29.5), HF 10 (9.5),<br>HTN 91 (86.7), COPD 19 (18.1), CVA/TIA 16 (15.2), CKD 3 (2.9), T2DM 34 (32.4), Previous vascular intervention 37 (35.2)
**Höbaus C et al., 2017 [40]**	Adherence to medical management and benefit the survival of PAD patients	Secondary care<br><br>Longitudinal prospective follow-up study	n=207<br><br>Gender<br>Intervention	Intervention<br>69.1 ± 10.6<br><br>Control	Stable patients with PAD	Intervention<br>CAD 117 (32), MI 60 (16),<br>Carotid artery disease 145 (39), HTN 340 (92),<br>Hypercholesterolaemia 359 (97), T2DM 108 (29.2)

			245 (66)<br><br>Control<br>206 (62)	66.7 ± 12.4		Control<br>HTN 256 (77), Hypercholesterolaemia 276 (83), T2DM 111 (33.4)
**Hussain MA et al., 2016 [41]**	Composite risk ratio of death, acute MI, or ischemic stroke	Secondary care<br><br>Observational cohort study	n=791<br><br>Gender<br>Intervention<br>180 (62.1)<br><br>Control<br>316 (63.1)	Intervention<br>67.9 ± 10.4<br><br>Control<br>68.2 ± 11.2	Symptomatic PAD, post vascular intervention /surgery *	Intervention<br>HF 21 (7.2), Stroke 16 (5.5), MI 19 (6.6), CAD 58 (20), COPD 11 (3.8), CKD 24 (8.3), Diabetes 55 (19)<br><br>Control<br>HF 62 (12.4), Stroke 28 (5.6), MI 49 (9.8), CAD 111 (22.2), COPD 18 (3.6)<br>CKD 63 (12.6), Diabetes 123 (24.6)
**Matthews S et al., 2016 [42]**	Not reported	Primary care<br><br>Audit	N=54<br><br>Gender not reported	Not reported	Symptomatic PAD with no recent intervention.	Not reported
**Rosenberg Y & Behrendt CA., 2023 [43]**	Feasibility of the intervention	Secondary care<br><br>Single centre pilot RCT	n=40<br><br>Gender<br>Intervention<br>12 (60)<br><br>Control<br>15 (75)	72.5 ± 9.0<br><br>Intervention<br>74.2 ± 8.5<br><br>Control<br>70.9 ± 9.5	Symptomatic patients with PAD of all stages who have undergone vascular intervention /surgery *	Intervention<br>IC 9 (45), CLTI 11 (55), Previous revascularisation intervention 18 (90),<br>Previous minor amputation 1 (5), Any diabetes 4 (20), Diabetes under pharmacological treatment 2 (10), Kidney failure 7 (35), Dialysis 1 (5), CAD 10 (50), MI 6 (30), HF 3 (15), Current cardiac dysrhythmia 3 (15), Current or former cardiac dysrhythmia 5 (25),<br>HTN 18 (90)<br><br>Control<br>IC 6 (30), CLTI 14 (70), Previous revascularisation intervention 13 (65), Previous minor amputation 3 (15), Any diabetes 7 (35), Diabetes under pharmacological treatment 7 (35), Kidney failure 8 (40), Dialysis 4 (20), CAD 6 (30), MI 4 (20),<br>HF 2 (10), Current cardiac dysrhythmia 5 (25), Current or former cardiac dysrhythmia 7 (35), HTN 17 (85)
**Siercke M et al., 2021 [37]**	Maximum walking distance at six months	Primary &secondary care<br><br>RCT	n=118<br><br>Gender<br>Intervention<br>23 (50)<br><br>Control<br>21 (44.6)	Intervention<br>70.5 ± 7.0<br><br>Control<br>70.1 ± 7.3	Medically managed patients with PAD who have not had a previous vascular intervention /surgery *	Intervention<br>Diabetes 11 (19),<br>MI 9 (15), Congestive HF 6 (10),<br>CVA/TIA 7 (12),<br>COPD 8 (14), Psoriasis 2 (3), Prolapsed disc 7 (12), Atrial flutter/fibrillation 5 (8), Spinal stenosis 3 (5), Osteoporosis 3 (5),<br>Back pain, undiagnosed 2 (3), Osteoarthritis 6 (10), HTN 44 (75)<br><br>Control<br>Diabetes 7 (12), MI 9 (15), Congestive HF 6 (10),

						CVA/TIA 8 (14), COPD 14 (24), Psoriasis 5 (8)<br>Prolapsed disc 7 (12),<br>Atrial flutter/fibrillation 5 (8), Spinal stenosis 4 (7),<br>Osteoporosis 4 (7), Back pain, undiagnosed 1 (2),<br>Osteoarthritis 9 (15), HTN 42 (71)
**Sutton M et al., 2018 [44]**	Reduce risk factors for PAD progression by improving blood sugar levels,<br>blood pressure control, keeping patients on moderate- or high-intensity statins, and<br>tobacco cessation or reduction.	Primary care (private cardiology practice)<br><br>Quality Improvement / prospective observational study	n=24<br><br>Gender<br>10 (41.7)	66.92 ± 8.75	Patients with PAD who required endovascular intervention	HTN 17 (70.8)<br>T2DM 12 (50)
**Davins Riu M et al., 2018 [45]**	Walking distance, Fontaine classification change, number of surgical interventions and healthcare resources use	Secondary care<br><br>Pilot RCT - pragmatic, parallel, randomized controlled trial	n=150<br><br>Gender<br>Intervention<br>68 (90.7)<br><br>Control<br>68 (90.7)	Intervention<br>>70 years old 28 (46.7)<br><70 years old 47 (52.2)<br><br>Control:<br><70 years old 43 (47.8)<br>>70 years old 32 (53.3)	Patients with IC symptoms	Intervention<br>Diabetes 31 (41.3), HTN 60 (80)<br><br>Control<br>Diabetes 34 (45.3), HTN 58 (77.3)
**Hatfield J et al., 2008 [46]**	Cardiovascular risk profile and quality of life	Secondary care<br><br>Service evaluation / prospective observational study	N=78<br><br>Gender<br>51 (65.4)	median age, 65 years (IQR: 56–74 years)	Patients with newly diagnosed IC	Diabetes with a HbA1c > 7.0%: 6 (11.7)<br>HTN 44 (56.4)
**Elfghi M et al., 2021 [47]**	Intervention effectiveness in achieving treatment goals for PAD risk factors	Primary & secondary care<br><br>Protocol for a randomised, parallel group, active-control trial	Target recruitment: 208 patients (104 per arm)<br><br>Gender<br>N/A - protocol	N/A - protocol	Symptomatic PAD	N/A - protocol
**Lovell M et al., 2011 [49]**	Intervention effectiveness on cardiovascular risk factor management	Secondary care<br><br>Quasi-experiment (before-after) study	n=103<br><br>Gender<br>63 (61)	58	Symptomatic PAD	MI 17 (16.7)<br>Diabetes 35(34)
**Fallon et al., 2024 [48]**	Intervention effectiveness on walking outcomes, quality of life and cardiovascular risk factors	Secondary care<br><br>Prospective, observational, single centre study	n=114<br><br>Gender<br>88 (77.2)	median age 66 years (IQR: 59.80-70.00 years)	Symptomatic PAD, post-vascular intervention /surgery *	HTN 81 (71.1)<br>T2DM 29 (25.4)

Table 3. Selection criteria of included studies

Study	Selection criteria	
	**Inclusion**	**Exclusion**
**Coca-Martinez M et al., 2023 [35]**	PAD patients (>50 y.o) affected by moderate to severe intermittent claudication as evidenced<br>1) clinically by functional limitations in walking activity and symptoms consistent with IC;<br>2) anatomically, by imaging evidence of stenosis below the inguinal ligament; and<br>3) hemodynamically, by an ABI ≤0.80 of the index leg	1)ischemic rest pain, ulceration or gangrene in the index leg<br>2) significant lesion(s) affecting the common iliac or the external iliac vessels<br>3) planned surgical bypass<br>4) urgent or emergent open surgery repair<br>5) prior interventions on the index leg<br>6) unable to speak English or French<br>7) premorbid conditions that precluded exercise and fitness assessments
**Mendez CB et al, 2019 [38]**	PAD diagnosis	N/A - intervention development
**Cucato G et al 2022 [39]**	1) Diagnosis of PAD confirmed by ABI <0.90 in one or both limbs<br>2) age>=40<br>3) Able to walk distance >50m,<br>4) Live in an area deemed in lowest 30% of super output area from Office of National Statistics	1) CLTI<br>2) short claudication distance <50m<br>3) severe heart disease (Grade III or IV, New York Heart Association)<br>4) severe ischemic or haemorrhagic stroke or neurodegenerative diseases<br>5) severe HTN (systolic blood pressure of more than 180 mm Hg, and diastolic blood pressure of more than 100 mm Hg)<br>6) uncontrolled cardiac disease (presence of complex arrhythmias, unstable angina during the previous month and myocardial infarction during the previous month)<br>7) a resting heart rate of more than 120 beats per minute<br>8) has already undergone angioplasty, bypass or other surgical intervention for PAD<br>9) other severe comorbid conditions preventing the ability to engage in physical activity inability or unwillingness to undertake the commitments of the study
**Haile ST et al., 2023 [36]**	1) age at least 18 years<br>2) Diagnosed with IC<br>3) absence of signs of CLTI<br>4) ability to speak and understand the Swedish language	1) dementia<br>2) planned discharge to a nursing home<br>3) not being accountable for administrating own medications<br>4) survival expectancy of less than 1 year
**Höbaus C et al., 2017 [40]**	1) stable PAD (ABI <0.9, Fontaine stage I–II, Rutherford stage I–III)<br>2) age between 40 and 90 years.	1) serum creatinine >0.265 mmol/L<br>2) connective tissue disease<br>3) hormone replacement therapy<br>4) critical illness within the last 6 months<br>5) type 1 diabetes mellitus<br>6) rest pain

		7) ulcers,<br>8) gangrene<br>9) known cancer
**Hussain MA et al., 2016 [41]**	Patients with symptomatic PAD, defined as one of the following:<br>1) a clinical history of intermittent claudication, ischemic rest pain, or tissue loss in conjunction with an ankle brachial index of 0.9 or less<br>2) previous peripheral arterial bypass surgery<br>3) previous percutaneous transluminal angioplasty (PTA) with or without a stent<br>4) a lower limb amputation for ischemia.	1) Patients seen by vascular surgeons at their institution-affiliated hospitals<br>2) Patients with atherosclerotic disease who were younger than 40 years
**Matthews S et al., 2016 [42]**	1) Patients with claudication symptoms limiting their everyday activity<br>2) Patients who have not had recent surgical intervention.	1) Asymptomatic PAD patients<br>2) recent vascular surgery intervention<br>3) unstable angina<br>4) unconrtolled cardiac arrhythmias,<br>5) CLTI<br>6) decompansated HF<br>7) severe or symptomatic valvular heart disease
**Rosenberg Y & Behrendt CA., 2023 [43]**	1)adult patients (≥18 years)<br>2) symptomatic stages of PAD, including IC and CLTI<br>3) underwent inpatient treatment at the study center,<br>4) undergoing endovascular or surgical procedures as well as amputations and diagnostic angiographies were included,<br>5) the possession of a phone to be contacted during the pilot study	1) Patients who were not capable of giving consent (e.g. dementia),<br>2) who were limited in the German language (a total of five patients during our recruitment process) or<br>3) patients hospitalised in an intensive care unit
**Siercke M et al., 2021 [37]**	1) conservatively treated patients with newly diagnosed IC using clinical assessment and ankle brachial index<br>2) age > 18 years<br>3) speaks and understands Danish<br>4) able to provide informed written content<br>5) citizens of one of eight municipalities of Greater Copenhagen belonging to the local healthcare centre<br>6) expected to be able to manage transportation<br>7) perform exercise	1) Failure to understand and cooperate according to the trial instructions<br>2) comorbidity complicating physical activity and exercise training<br>3) lack of informed consent
**Sutton M et al., 2018 [44]**	1) patients with PAD who required endovascular intervention<br>2) the ability of the subject to read and write	1) patients with inability to ambulate<br>2) unstable angina<br>3) decompensated HF<br>4) uncontrolled cardiac arrhythmias<br>5) severe or symptomatic valvular heart disease<br>6) who were unable to read or write.
**Davins Riu M et al., 2018 [45]**	1) intermittent claudication confirmed by a vascular surgery specialist according to clinical, physical, and hemodynamic criteria<br>2) aged 18 years or older<br>3) signed informed consent<br>4) access to the Internet by the patient or a relative<br>5) fluency in the Catalan and/or Spanish language.	1) serious psychiatric disorder or cognitive impairment before consideration for the study<br>2) previous patent arterial surgery<br>3) severe chronic obstructive pulmonary disease (GOLD stage III/IV)<br>4) severe congestive heart failure (NYHA class III/IV)<br>5) active malignant disease with expected survival of less than 1 year

**Hatfield J et al., 2008 [46]**	Newly diagnosed PAD patients with IC	No specific exclusion criteria.<br>However they report that 23 patients had coexisting coronary heart disease and were excluded from this analysis.
**Elfghi M et al., 2021 [47]**	1) ≥18 years<br>2) PAD: diagnosed by at least one of the following a)Ankle-brachial index of less than 0.90 in at least one lower extremity, b)Toe brachial index of less than 0.60, c)Evidence of arterial occlusive disease in one lower extremity detected by duplex ultrasonography, computed tomographic angiography, or magnetic resonance angiography<br>3)Symptomatic PAD (Rutherford category 2 and above)<br>4) Patients should have at least one of the following risk factors: a)Blood pressure > 140/80 mmHg, b)Fasting blood sugar (FBS) > 53 mmol/mol, c)Glycosylated haemoglobin (HbA1c) > 7%, d)Total cholesterol > 5 mmol/L, e)Low-density lipoprotein (LDL) cholesterol > 2.6 mmol/L, f)Triglycerides > 1.7 mmol/L, g)High-density lipoprotein (HDL) < 1.0 mmol/L in men and < 1.2 mmol/L in women, h)Physical activity less 30 min for 5 days per week, i)Body mass index (BMI) > 25 kg/m2, j)Waist circumference > 80 cm in women, and > 94 cm in men, k)Current smoker or exposure to tobacco in any form, l)Unhealthy diet, Mediterranean diet score less than 10 points	1) Involvement in another clinical trial in the previous 6 months<br>2) Legal incapacity<br>3) Inadequate English language ability to understand the content of the intervention programme<br>4) Significant cognitive impairment or mental illness<br>5) Refusal to participate in a certain part of the intervention<br>6) Patient suffering from a comorbidity that could affect either their physical participation in the intervention arm or influence outcomes (e.g. ischemic heart failure or severe chronic kidney disease with an estimated glomerular filtration rate of less than 30 mL/min)<br>7) Patient is immobile<br>8) Contraindication to anticoagulation and antiplatelet medications or any of the risk factors treatment.
**Lovell M et al., 2011 [49]**	1)PAD diagnosis<br>2) PAD patients who were not on antiplatelet therapy<br>3)PAD patients who had suboptimal blood pressure control and elevated lipid profiles	Not reported
**Fallon et al., 2024 [48]**	1)An ABI of ≤90 in either leg, or previous surgery for PAD<br>2)ongoing exertional leg symptoms	CLTI, including pain at rest, non-healing ulceration or gangrene (Fontaine Classification III and IV)

Abbreviations

ABI: ankle brachial index; CLTI: chronic limb threatening ischemia; IC: Intermittent claudication; PAD: Peripheral artery disease

Table 4. TIDIER – Intervention characteristics

Study	Intervention name	Theory & Rationale	Description	Behaviour targeted	Materials	Provider	Mode of delivery & Location	Duration/ time-points	Tailoring	Intervention modification
**Coca-Martinez M et al., 2023 [35]**	N/A	No theory/ framework	Multimodal multidisciplinary prehabilitation programme that was delivered prior to	Smoking cessation, Exercise, Diet	1) Treadmill,<br>2) elastic bands,<br>3)blood pressure monitor,	Multidisciplinary healthcare professionals, including physicians,	Combination of F2F, hospital-based and home-based. SET was group-based	12 weeks prehabilitation programme.<br>-SET once per week and	Yes- The intervention was tailored for exercise (according to	Not reported

			vascular surgical intervention using the following components: i) weekly supervised exercise session; ii) home-based exercise prescription; iii) nutritional counselling and supplementation; iv) smoking cessation therapy; and v) psychosocial support.		4) exercise logbook,<br>5) Patient Generated Subjective Global Assessment (PG-SGA),<br>6) 3-day food diary,<br>7) Fagerstrom Test for Nicotine Dependence,<br>8) relaxation audio,<br>9) SET,<br>10) blood pressure monitor	kinesiologists, dieticians, psychologists and/or smoking cessation specialists.<br><br>The intervention also included elements of patient self-management.	and delivered in hospital.	lasted a total of 1 hr.<br>-Home based advised to walk for at least 30–40 min at moderate intensity 3 times/week & muscle training 3times/week.<br><br>Assessments: Baseline, 3,6 and 12 months follow up.	their baseline functional capacity results), diet (based on the dietary status and conditions of the patient) and psychological intervention (based on the psychological status).	
**Mendez CB et al, 2019 [38]**	N/A	No theory used. Framework: Contextualized Instructional Design (CID) model.	Educational, app-based intervention that also allows the exchange of messages between the patient & HCP.	Medication adherence, Exercise	1)Access to an IOS or Android smartphone and WIFI/Cellular data. 2)App that was developed for the ANDROID and IOS platforms. For its installation, it is necessary to setup to allow the installation from stores (play store and Apple store) or through availability by the health service.	Self-managed by the patients. A HCP is available for questions that can be requested by the patients through the app.	App/ Virtual. Home-based	N/A	Not reported	N/A – intervention development
**Cucato G et al 2022 [39]**	Textpad	No theory/framework used. Co-designed intervention. Specific barriers to SET they are trying to target are financial limitations and travel distances.	A home-based, virtual exercise and an individual educational lifestyle programme.	Smoking cessation, Exercise, Diet & alcohol	1)Mobile/smart phone device,<br>2) internet connection (provided by the research team)<br>3)Zoom app,<br>4) Educational materials and videos	Elements of patient self-management, plus delivery by health trainers	Remote/Telehealth – Virtual. Home-based	12 weeks intervention. Exercise programme twice/week, lifestyle programme once/week	Yes- Elements of tailored plan and shared decision making	N/A – protocol

**Haile ST et al., 2023 [36]**	FASTIC	No theory used. Framework: Medical Research Council (MRC) guidelines for developing and evaluating complex interventions. Model of care pathway used: the Gothenburg person-centred care (PPC) model.	Nurse-led, person-centred, health-promoting programme, which includes the establishment of a partnership between the professional healthcare worker and the patient; patient narratives; and a documented self-care plan with goals, self-care activities, and a plan for future follow-up and revision.	Medication adherence, Smoking cessation, Exercise	1)training provided to nurses in motivational interviewing and PPC<br>2) mediums for information i.e. movies,<br>3) printed information sheets, 4) referrals to support groups	Vascular nurse specialists trained in PCC and in motivational interviewing	Combination of remote phone calls and F2F appointments. Hospital and home-based	1 year after surgical treatment. Three visits at the outpatient clinic and two telephone calls.	Yes – self-care plan, and discussions were personalised to suit individual needs	Not reported. When hand searched the protocol and the RCT paper, no modification were identified.
**Höbaus C et al., 2017 [40]**	N/A	No theory/framework	A secondary care, vascular medicine centre based medical management intervention.	Medication adherence, Smoking cessation, Exercise	1)Letters with the updated medication regime sent to the treating primary care physician at each visit<br>2) letters to relay information to participants,<br>3) self-monitoring blood pressure machine,<br>4) monetary resources for non-generic drugs.	Vascular surgeon and vascular specialist technicians.	F2F. Hospital-based	Patients were followed up for a 5 year period and had an appointment every 6 or 12 months.	N/A	Not reported
**Hussain MA et al., 2016 [41]**	N/A	No theory/framework	A programme that focuses on guideline-recommended cardiovascular risk-reduction therapies, including: antiplatelet agents, statins, angiotensin-converting enzyme inhibitors, blood pressure control, lipid control, diabetic glycemic control, smoking cessation, and target body mass index	Medication adherence, Smoking cessation	1)Educational material and leaflets for GPs about clinical practice guidelines on PAD risk management to family physicians of patients in the SAVR program, and specific suggestions for	Vascular nurse specialist and vascular surgeon	F2F. Hospital-based	Not reported - data collected from July 1, 2004, to April 30, 2007	N/A	Not reported

					risk management. 2) Informational letters informing patients about their specific risk factors, and management strategies.					
**Matthews S et al., 2016 [42]**	N/A	No theory/framework	A community-based podiatry-led lower limb arterial assessment service and a cardiac rehabilitation supervised exercise programme.	Medication adherence, Smoking cessation, Exercise advice, Healthy diet & weight management	An established cardiac rehabilitation team.	Cardiac rehabilitation staff and vascular triage team	F2F. Hospital-based	12 weeks cardiac rehabilitation.	Yes- Individualised exercise plan	Not reported
**Rosenberg Y & Behrendt CA., 2023 [43]**	N/A	No theory/framework	An educational structured risk factor management intervention and supervised exercise.	Medication adherence, Smoking cessation, Exercise, Diet	1)Questionnaires, 2)oral education, 3) a copy of the brochure and a list of contact data for SET in Hamburg, Germany, 4) SET centres, 5) trained staff to administer educational sessions, telephones.	Trained final year medical student about best medical therapy for symptomatic patients with PAD. A supervisor was also present if necessary.	Combination of F2F (for the assessment) and home-based (for those doing home-based exercise)	10 weeks. The intervention was given twice during hospitalisation following vascular intervention. Post-hospitalisation: One planned telephone contact per week.	Not reported	Not reported
**Siercke M et al., 2021 [37]**	The CIPIC Rehab	No framework. Theory: Social Cognitive-Behavioral Theory.	A cross sector (community/hospital) exercise and lifestyle intervention, cardiac rehabilitation programme in a community based setting for patients with intermittent claudication (IC).	Smoking cessation, Exercise, Diet	1)Pedometer 2) logbook, 3)circuit training and strength training equipment	Multidisciplinary approach including cardiac physiotherapists, nurses and dietitians.	F2F and remote (text-messages). Community rehab based and home-based	12 weeks. 2 weekly exercise sessions. Study follow-up: 6 and 12 months.	Yes- personalised follow-up texts	Not reported. When hand searched the protocol and the RCT paper, no modification were identified.
**Sutton M et al., 2018 [44]**	N/A	No theory/framework	A nurse-led and doctor-led clinical follow-up risk factor management programme	Medication adherence, Smoking cessation, Exercise	1)Handouts in V3 for risk factor modification care plan,	Cardiologist and nurse practitioner	F2F. Clinic/practice based.	90 days. 4 visits in total.	Yes- customised risk modification plan	Not reported

					2) Cleveland Clinic ‘‘PAD Walking Program’’, so an area to walk or a treadmill.					
**Davins Riu M et al., 2018 [45]**	Control Telehealth Claudication Intermittent (CONTECI)	No theory/framework	A virtual educational and self-management intervention	Medication adherence, Smoking cessation, Exercise	1)Internet access, 2) computer or mobile device (smart phone or tablet), 3) intervention app	Self-managed by the patient via the app, with stand-by healthcare professional input	Combination of F2F, hospital-based (for initial assessment and any follow ups needed) and remote (app)	12 months study follow up. 3 months follow ups	Yes- Personalised advice depending on how they answer the questions	Not reported
**Hatfield J et al., 2008 [46]**	N/A	No theory/framework	Nurse-led risk management programme	Medication adherence, Smoking cessation	Exercise regimen provided to patients.	Vascular nurses and patient self-management. Referral to specialist nurses (e.g. diabetes) when required.	F2F. Hospital based	3 months. Patients had a single nurse assessment at baseline and 3 months	N/A	Not reported
**Elfghi M et al., 2021[47]**	N/A	No theory/framework	A nurse-led, community-based, lifestyle and risk factor modification and exercise intervention	Medication adherence, Smoking cessation, Exercise, Diet	1)food diary, 2)pedometer, 3)exercise class and educational workshops, 4)basic equipment for measures (blood, BMI, glucose, lipid)	Multidisciplinary approach including nurses, dieticians, and exercise specialists	Combination of F2F, hospital based (for assessments) and F2F community, group- based, (for intervention)	12 weeks intervention. Visit: baseline, 12 weeks and 1 year. Weekly classes.	Yes- individualised assessment with goal planning	N/A – protocol. They will report in the main paper any modifications occurred.
**Lovell M et al., 2011 [49]**	N/A	No theory/framework. Model of care pathway used: Chronic Care Model (CCM)	A multi-disciplinary team approach for cardiovascular risk factor management and modification using elements of the CCM.	Medication adherence, Smoking cessation, Diet	1)Equipment for clinic and dedicated clinic space; 2)Educational materials (pamphlets and information sheets) 3)Patients graphs depicting their cholesterol levels, blood pressure, A1C levels and overall risk-assessment score given to	Multidisciplinary approach including vascular nurse trained in using elements of the CCM, dieticians, vascular surgeons and family physicians	F2F. Hospital based	Average follow-up time was 528 days. Patients were assessed initially and at regular follow-up periods (3 – 6 months)	Yes – personalised based on patient’s needs and clinical factors (e.g cholesterol adapted graphs)	Not reported

					patients at each visit, 4) Scientifically proven education materials from vascular societies, 5) Vascular risk management database containing all patient and clinical information including labs and medications; 6) Electronic patient charts with all previous hospital visits and diagnostic tests, 7) Follow up letters sent to the referring vascular surgeon and family physicians.					
**Fallon et al., 2024 [48]**	N/A	No theory/framework.	A standalone PAD rehabilitation programme, involving two individualised exercise sessions, a group education session and other individual interventions to control risk factors per week.	Medication adherence, Smoking cessation, Exercise, Diet & alcohol	1)outpatient gymnasium 2)exercise equipment including treadmill, bikes, rowers, stairs and cross trainers, and weights, 3)basic equipment for measures (not specified), 4) BP monitor	Multidisciplinary approach including nurses, dietitians, physiotherapists	F2F, hospital based. Group based for education session.	10 weeks intervention delivered twice a week.	Yes- personalised risk factor and medication adherence management, as well as individualised exercise sessions.	Not reported

Abbreviations:

F2F: Face to face; PAD: Peripheral artery disease; RCT: Randomised control trial; SET: Supervised exercise therapy

Table 5. Targeted behaviours, BCTs and intervention effectiveness

Study	Study design	Number of participants	Behaviour targeted	BCTs	Intervention effect on adherence	Intervention effect on clinical outcomes
**Coca-Martinez M et al., 2023 [35]**	Pilot RCT - single-blind, 2-arm, parallel, pilot-RCT	24<br><br>Intervention<br>12<br><br>Control<br>12	Smoking cessation,<br>Exercise,<br>Diet	1) Instructions on how to perform the behaviour (through education),<br>2) information about health consequences,<br>3) Social support (emotional) (psych component),<br>4) Social support (unspecified) (from HCPs),<br>4) Biofeedback (using logbooks, CO test for smoking),<br>5) Self monitoring of behaviour (patients record in diary),<br>6) Demonstration of the behaviour	2 patients (40% of the active smokers) successfully stopped smoking [a]<br><br>Adherence to each prehabilitation component was 83% for in hospital supervised exercise, 90% for home-based exercise and 69% for nutrition [a]	Change in functional and walking capacity between intervention vs. control at 12 weeks results, mean (SD):<br><br>6 Min Walk Test distance: 60 (74) vs. -11 (40) meters, **p<0.05**<br><br>Claudication Onset distance: 48 (79) vs. −26 (65), p=0.64<br><br>Walking Impairment Questionnaire:<br>15.6 (13.0) vs −1.7 (21.7), **p<0.05**
**Mendez CB et al, 2019 [38]**	Intervention development	N/A – intervention development	Medication adherence,<br>Exercise	1) Instructions on how to perform the behaviour (through education),<br>2) Self monitoring of behaviour,<br>3) Prompts and cues (reminders to enter info),<br>4) Conserving mental resources (food recipes)	N/A – intervention development paper	N/A – intervention development paper
**Cucato G et al 2022 [39]**	Protocol for a pilot RCT	Target recruitment: 60	Smoking cessation,<br>Exercise,<br>Diet & alcohol	Potential BCTs:<br>1) Instructions on how to perform a behaviour	N/A – protocol	N/A – protocol

				2) Goal setting (behaviour),<br>3) Problem solving,<br>4) Self-monitoring of behaviour,<br>5) Social support (unspecified),<br>6) Social support (practical),<br>7) Information about health consequences,<br>8) Comparative imagining of future outcomes<br>9) Demonstration of the behaviour		
**Haile ST et al., 2023 [36]**	Non-blinded RCT	204<br><br><u>Intervention</u><br>99<br><br><u>Control</u><br>105	Medication adherence, Smoking cessation, Exercise	1) Goal setting (behaviour),<br>2) Social support (emotional),<br>3) Information about health consequences,<br>4) Instruction on how to perform a behaviour,<br>5) Comparative imagining of future outcomes,<br>6) Verbal persuasion about capability,<br>7) Self-monitoring of behaviour,<br>8) Feedback on behaviour,<br>9) Review of behavioural goals	Intervention vs. control at 1 year:<br><br><u>Statin adherence:</u> 79% vs. 82%, p=0.464,<br><br><u>Antiplatelet adherence:</u> 92% vs. 91%; p=0.700<br><br><u>Ex-smokers:</u> 78 (80%) vs. 79 (77.5%), p=0.734<br><br><u>Current smokers:</u> 12 (12%) vs. 15 (14.7%), p=0.544	12 months results Intervention vs Control, n(%):<br><br><u>LDL</u><br>Patients within treatment goal (≤ 1.8 mmol/l): 36 (41) vs. 39 (43), p= 0.880<br><br>Deteriorated from treatment goal (n = 44|47): 20(46) vs. 12 (28), p=0.086<br><br><u>Total cholesterol</u><br>Patients within treatment goal (≤ 5 mmol/l): 70(78) vs. 81(87), p=0.186<br><br>Deteriorated from treatment goal (n =75|79): 12(16) vs. 4(5), **p=0.034**<br><br><u>HbA1c</u><br>Patients within treatment goal (≤ 52 mmol/mol with diabetes, ≤ 46 mmol/mol without diabetes) (n =81|84): 57(70) vs. 71(85), **p=0.040**<br><br>Reached treatment goal (n = 26|14): 6(23) vs. 5(36), p=0.469<br><br><u>Systolic blood pressure (n = 93|98)</u><br>Patients within treatment goal (< 140 mmHg): 39(42) vs. 32(33), p=0.231<br><br>Reached treatment goal (n = 55|69): 19(35) vs. 16(23), p=0.228

						Diastolic blood pressure (n = 93|98)<br>Patients within treatment goal (< 90 mmHg): 87(94) vs. 89(91), p=0.594<br><br>Reached treatment goal (n = 9|7): 8(89) vs. 3(43), p=0.106<br><br>Pain-free walking distance<br>Patients with unlimited pain-free walking distance at 1 year after surgery (n = 93|95): 61(66) vs. 56(59), p=0.370<br><br>Deteriorated pain-free walking distance at 1 year after surgery (n = 55|71): 12(22) vs. 21(30), p=0.415<br><br>ABPI<br>Change (1 year – baseline), median (i.q.r.): 0.30 (0.14 to 0.42) vs. 0.27 (0.13 to 0.43), p=0.146<br><br>BMI<br>Change (1 year – baseline), median (i.q.r.): −0.29 (−0.73 to 0.34) vs. 0.00 (−0.35 to 0.91), p=0.869<br><br>Waist circumference (cm)<br>Change (1 year – baseline), median (i.q.r.): −2 (−6 to 1) vs. −1 (−6 to 2), p=0.471
**Höbaus C et al., 2017 [40]**	Longitudinal prospective follow-up study	702<br>Intervention<br>370<br><br>Control<br>332	Medication adherence, Smoking cessation, Exercise	1) Review of behavioural goals,<br>2) Feedback on behaviour,<br>3) Biofeedback<br>4) Information about health consequences	Statins use increased from 48.8% (baseline) to 89% (6 months). [a]<br><br>Antiplatelet medication use increased from 88.7% (baseline) to 90.9% (6 months). [a]<br><br>ACE inhibitors/ARB use ranged from 74.3% (baseline) to 86.1% (6 months). [a]<br><br>Active smoking declined from 34.7% (baseline) to 24.4% (6 months) [a]	At the end of follow up, survival in the intervention group was 90.8% vs 66% in the control group (absolute CI: 18.96–30.73%; 37.6% relative) [a]<br><br>Mortality was reduced from 82 to 21 events per 1000 patient-years resulting in a rate ratio is 0.26 for intervention group. In the control group, all-cause mortality was increased by a HR of 3.7 (95% CI: 2.51–5.46, **p<0.001**).<br><br>The intervention group led to the identification of Intensified screening by combination of 48% (n = 176) patients with T2DM, **p<0.001**.
**Hussain MA et al., 2016 [41]**	Observational cohort study	791<br><br>Intervention<br>290	Medication adherence, Smoking cessation	1) Instructions on how to perform the behaviour,<br>2) Feedback on behaviour,	Baseline vs end of follow up [+]<br><br>Antiplatelet use (67% vs 80%; **p= 0.03**),	Patients in the intervention group experienced lower mortality (adjusted HR, 0.60; 95% CI, 0.48-0.75, **p<0.001**) than control patients.

		Control<br>501		3) Information about health consequences	Statin use (67% vs 88%; **p< 0.001**),<br><br>ACE inhibitor or angiotensin receptor blocker use: 58% vs 72%, **p=0.02**,<br><br>Smoking cessation: 75% vs 88%, **p<0.001**	Patients in the intervention group were also less likely to undergo major amputation (adjusted HR, 0.47; 95% CI, 0.29-0.77, **p=0.002**), minor amputation (adjusted HR, 0.26; 95% CI, 0.13-0.54, **p<0.001**), or arterial bypass surgery (adjusted HR, 0.47; 95% CI, 0.30-0.73, **p<0.001**).<br><br>Patients in the intervention group were less likely to be hospitalized due to heart failure: adjusted HR, 0.73; 95% CI, 0.53-1.00, p=0.048<br><br>Blood pressure control: baseline 35% vs end-of-follow-up 52%, **p=0.02**,<br><br>LDL cholesterol control: baseline 63% vs end-of-follow-up 86%, **p=0.002**,<br><br>Glycaemic control: baseline 44% vs end-of-follow-up 52%, p=0.53,<br><br>Target BMI: baseline 27% vs end-of-follow-up 38%; **p=0.049**
**Matthews S et al., 2016 [42]**	Audit	54	Medication adherence, Smoking cessation, Exercise advice, Healthy diet & weight management	1) Monitoring of behaviour by others without feedback,<br>2) Instruction on how to perform the behaviour,<br>3) Social support (emotional)	45 patients (84%) were on best medical therapy of anti-platelet and lipid-regulating drugs at the completion of the programme, [a]<br><br>7 patients (13%) quit smoking, [a]<br><br>10 patients (18.5%) had reduced the number of cigarettes they smoke [a]<br><br>7 patients (13%) joined a gym, [a]<br><br>4 patients (7%) reported a weight reduction [a]	Of the 54 patients who completed the programme, 39 (72%) reported an improvement in their intermittent claudication symptoms, 13 (24%) reported no change and 2 (4%) felt their symptoms had deteriorated. [a]
**Rosenberg Y & Behrendt CA., 2023 [43]**	Single centre pilot RCT	40<br><br>Intervention<br>20<br><br>Control:	Medication adherence, Smoking cessation, Exercise, Diet	1) Instructions on how to perform the behaviour,<br>2) Social support (unspecified),	End of follow up data intervention vs. control:<br><br>Medication adherence:<br>100% in both groups [a]	End of follow up data intervention vs. control:<br><br>Reached a normal weight BMI:<br>0% vs. 6.7% [a]

		20		3) Monitoring of outcome(s) of behaviour by others without feedback,<br>4) Demonstration of the behaviour	<u>Smoking cessation:</u><br>0% vs. 13.3% [a]<br><br><u>Reduction in smoking consumption:</u><br>6.3% vs. 13.3% [a]<br><br><u>SET attendance:</u><br>0% in both groups [a]<br><br><u>Home-based exercise:</u><br>62.5% vs. 40% [a]<br><br><u>Achieved exercise goals:</u><br>31.3% vs. 20% [a]	<u>Weight reduction but still overweight or obese (BMI >25 kg/m2):</u><br>25% vs 33.3% [a]<br><br><u>Major adverse limb event:</u><br>25% vs. 6.7% [a]<br><br><u>Major adverse cardiovascular event</u>: 0% for both groups [a]
**Siercke M et al., 2021 [37]**	RCT	118<br><u>Intervention</u><br>46<br><br><u>Control</u><br>47	Smoking cessation, Exercise, Diet	1) Instructions on how to perform the behaviour,<br>2) Social support (emotional),<br>3) Self-monitoring of behaviour,<br>4) Feedback on behaviour,<br>5) Biofeedback,<br>6) Information about health consequences,<br>7) Verbal persuasion about capability,<br>8) Goal setting (behaviour),<br>9) Comparative imaging of future outcomes,<br>10) Demonstration of the behaviour	Baseline vs. 6-months follow up, n(%)<br><br><u>Healthy diet:</u> Intervention 1(2)/ Control 0(0) vs. Intervention 5(11)/ Control 0(0) [a]<br><br><u>Physical activity:</u> Intervention 18(31)/ Control 21(36) vs. Intervention 24(52) / Control 15(32), 95% CI 1.66–18.82, **p=0.002**<br><br><u>Smoking cessation at 1 year:</u> 7 patients quit smoking in the intervention group compared to baseline, whilst no patients in the control group quit smoking (p=0.58).	Baseline vs. 6-months follow up, mean (SD):<br><br><u>MWD:</u> Intervention 248 (309.9) / Control 188 (300.7) vs. Intervention 350.5 (447.6) / Control 253 (325.4), 95% CI 1.10 – 1.70; **p=0.005**<br><br>Pain free walking distance: Intervention 115 (151.5) / Control 96.5 (157.9) vs. Intervention 133.5 (207.9) / Control 96 (165.8), 95% CI 0.99–1.65, p=0.060
**Sutton M et al., 2018 [44]**	Quality Improvement	24	Medication adherence, Smoking cessation, Exercise	1) Instructions on how to perform the behaviour,<br>2) Feedback on behaviour,<br>3) Self-monitoring of behaviour,<br>4) Goal setting (behaviour),<br>6) Review behaviour goal(s),	100% <u>statin prescription</u> [a]<br><br>2 participants (8.3%) <u>walked</u> for 31-40 times during the 90day intervention, and only 3 participants (12.5%) walked for >40 times during the 90day intervention. [a]<br><br>4 (57.1%) reported a reduction in their <u>tobacco</u> use [a]	Baseline vs. end of follow-up data, mean (SD):<br><br><u>Total cholesterol:</u> 180.17 (40.41) vs. 172.67 (40.52), p=0.066<br><br><u>LDL:</u> 98.29 (32.74) vs. 84.79 (33.64), **p=0.007**<br><br><u>Blood pressure</u> control goal as per guidelines: 14 (58.3%) patients vs. 21 (87.5%) patients [a]

				7) Information about health consequences		Six-minute walk (meters): 178.84 (116.08) vs. 232.44 (98.64), **p=0.030**
**Davins Riu M et al., 2018 [45]**	Pilot RCT - pragmatic, parallel, randomized controlled trial	150<br>Intervention<br>75<br><br>Control:<br>75	Medication adherence, Smoking cessation, Exercise	1) Instructions on how to perform the behaviour,<br>2) Self monitoring of behaviour,<br>3) Feedback on behaviour,<br>4) Information about health consequences	Intervention vs. control results, n(%):<br><br>Medication adherence at 6 months: 57(83.8) vs. 49 (69.0), **p=0.031**<br><br>Medication adherence at 12 months: 55 (75.3) vs. 50 (70.4), p=0.317<br><br>Smoking abstinence at 12 months: 19 (27.5) vs. 14 (20.3), p=0.892<br><br>Exercised more at 12 months: 15 (21.4) vs. 10 (14.7), p= 0.125	Intervention vs. control results at 12 months, n(%):<br><br>Claudication distance:<br>Less 5 (7.1) vs. 11 (15.9)<br>Same 45 (64.3) vs. 38 (55.1)<br>More 20 (28.6) vs. 20 (29)<br>p= 0.243<br><br>MWD (meters):<br><150: 2 (2.9) vs. 8 (11.6)<br>151-500: 12 (17.4) vs. 13 (18.8)<br>500-1,000: 15 (21.7) vs. 8 (11.6)<br>>1,000: 40 (58.0) vs. 40 (58.0)<br>p= 0.123<br><br>Requiring surgery:<br>1 (1.4) vs. 4 (5.6), p=0.182
**Hatfield J et al., 2008 [46]**	Service evaluation / prospective study	78	Medication adherence, Smoking cessation	1) Instructions on how to perform the behaviour,<br>2) Biofeedback,<br>3) Social support (unspecified)	Baseline vs. 3 months follow-up results, n(%)<br><br>Antiplatelet adherence: 68 (88.5) vs. 78 (100) [a]<br><br>Statin adherence: 52 (66.6) vs. 78 (100) [a]<br><br>Current smokers: 52 (66.6) vs. 43 (55.5) [a]	Baseline vs. 3 months follow-up results, n(%)<br><br>Diabetics with a HbA1c > 7.0%: 6 (50.0) vs. 5 (41.6) [a]<br><br>Hypertension (reading > 140/90 mm Hg): 44 (56.4) vs. 38 (48.7) [a]<br><br>Total cholesterol (mmol/L): 5.2 (4.5–6.1) vs. 4.5 (4.0–4.9), **p<0.001**<br><br>LDL (mmol/L): 3.1 (2.5–3.7) vs. 2.5 (2.1–2.9), **p<0.001**<br><br>Triglycerides (mmol/L): 1.7 (1.2–2.5) vs. 1.4 (1.0–2.3), **p=0.001**
**Elfghi M et al., 2021 [47]**	Protocol for a randomised, parallel group, active-control trial	Target recruitment: 208 patients (104 per arm)	Medication adherence, Smoking cessation, Exercise, Diet	1) Instructions on how to perform the behaviour,<br>2) Goal setting (outcome),<br>3) Review outcome goal(s) (e.g lipid improvement),	N/A – protocol	N/A – protocol

				4) Problem solving (Identifying barriers to exercise),<br>5) Self-monitoring of behaviour (pedometer and food diary),<br>6) Social support (unspecified),<br>7) Demonstration of the behaviour		
**Lovell et al., 2011 [49]**	Quasi-experiment (before-after) study	103	Medication adherence,<br>Smoking cessation,<br>Diet	1)Verbal persuasion about capability (for disease in general)<br>2) Goal setting (behaviour),<br>3) Goal setting (outcome),<br>4) Problem solving, 5) Instructions on how to perform behaviour,<br>6) Information about antecedents,<br>7) Monitoring outcomes of behaviour without feedback,<br>8) Biofeedback,<br>9) Feedback on outcomes of behaviour,<br>10) Social support (unspecified),<br>11) Prompts and cues,<br>12) Information about health consequences	Medication adherence rates were not reported<br><br>9 of the 58 patients (16%) who smoked at baseline successfully quit [a]	Baseline vs. end of follow up results, mean (SD):<br><br><u>BMI (kg/m^2):</u> 27.6 (5.1) vs. 27.9 (5.6), p=0.09<br><br><u>Systolic blood pressure (mmHg):</u> 143.3 (19.9) vs. 137.3 (16.1), **p<0.001**,<br><br><u>Diastolic blood pressure (mmHg):</u> 79.7 (10.9) vs. 75.8 (10.3), **p<0.001**,<br><br><u>LDL (mmol/L):</u> 2.89 (1.05) vs. 2.15 (0.67), **p<0.001**,<br><br><u>HDL (mmol/L):</u> 1.15 (0.35) vs. 1.10 (0.35), **p=0.0057,**<br><br><u>Total cholesterol (mmol/L):</u> 4.9 (1.1) vs. 4.0 (0.8), **p<0.001**,<br><br><u>Blood glucose mmol/L:</u> 6.26 (1.84) vs. 6.13 (1.13), p=0.44
**Fallon et al., 2024 [48]**	Prospective, observational, single centre study	114	Medication adherence,<br>Smoking cessation,<br>Exercise,<br>Diet & alcohol	1)Instructions on how to perform a behaviour,<br>2) Social support (practical),<br>3) Demonstration of the behaviour	Medication adherence rates were not reported<br><br>Non-smokers pre-programme 55 (62%) vs. post-programme 60 (68%), p=0.302	Baseline vs. end-of-programme results, median (Q1, Q3):<br><br><u>Systolic blood pressure (mmHg):</u> 137 (122, 147) vs. 134 (124, 143), p= **0.037**<br><br><u>Diastolic blood pressure (mmHg):</u> 76 (70, 86) vs. 73 (67, 81), **p=0.003**<br><u>Total cholesterol (mmol/L):</u> 4.30 (3.73, 5.00) vs. 3.90 (3.40, 4.28), **p<0.001**

						LDL (mmol/L): 2.30 (1.90, 2.98) vs. 1.95 (1.70, 2.30), **p<0.001**<br><br>HDL (mmol/L): 1.26 (0.99, 1.59) vs. 1.25 (1.03, 1.50), p=0.591<br><br>Triglycerides (mmol/L): 1.53 (1.00, 2.11) vs. 1.34 (0.96, 1.85), p=0.076<br><br>Glucose levels (mmol/L): 5.30 (4.90, 6.60) vs. 5.50 (5.20, 6.40), p=0.326<br><br>HbA1c levels (mmol/mol): 6.05 (5.60, 7.35) vs. 6.15 (5.63, 6.50), p=0.627<br><br>Weight (kg): 81.4 (71.3, 88.8) vs. 81.6 (70.8, 87.9), p=0.961<br><br>Waist circumference (cm): 95.8 (84.9, 101.9) vs. 99.5 (95.3, 108.8), p=0.073<br><br>BMI (kg/m^2): 28 (25.2-32.0) vs. 28 (25.0-31.0), p=0.493<br><br>Maximum claudication distance (metres): 250 (178, 373) vs. 700 (483, 840), **p<0.001**<br><br>Maximum claudication time (seconds): 298 (215, 432) vs. 795 (553, 960), **p<0.001**

[a] p-value not reported

[+] no specific follow-up time was provided; the median follow-up for the cohort was 5.3 years (interquartile range, 1.8-6.9 years).

Abbreviations:

BMI: Body mass index ; LDL: Low-density lipoprotein ; MWD: Maximum walking distance; T2DM: Type 2 Diabetes Melitus, RCT: Randomised control trial

Table 6. Behavioural Change Techniques used for each target behaviour

**Behavioural Change Techniques**	**Intervention targeted behaviours**			
	Medication adherence	Smoking cessation	Exercise	Diet
Goal setting (behaviour)	2*	4	5	4
Problem solving	0	0	1	0
Goal setting (outcome)	2	0	1	0
Review behaviour goals	2	3	3	1
Review outcome goals	1	0	0	0
Monitoring of behaviour by others without feedback	0	0	2	0
Feedback on behaviour	2	4	5	2
Self-monitoring of behaviour	5	3	8	4
Monitoring of outcome(s) of behaviour without feedback	0	0	1	1
Biofeedback	3	1	3	0
Social support (unspecified)	2	2	3	1
Social support (practical)	0	0	1	0
Social support (emotional)	1	1	2	1
Instructions on how to perform the behaviour	10	10	14	7
Information about health consequences	4	4	4	3
Demonstration of behaviour	0	0	6	0

Prompts & cues	1	0	1	0
Comparative imagining of future outcomes	1	2	1	1
Conserving mental resources (cook book)	0	0	0	1
Verbal persuasion about capacity	1	1	2	1

*The numbers represent the number of studies that have used the specific BCTs
